# NF-κB activation enhances cell death by antimitotic drugs in human prostate cancer cells

**DOI:** 10.1186/1476-4598-9-182

**Published:** 2010-07-09

**Authors:** Ricardo Parrondo, Alicia de las Pozas, Teresita Reiner, Priyamvada Rai, Carlos Perez-Stable

**Affiliations:** 1 Geriatric Research, Education, and Clinical Center and Research Service, Bruce W. Carter Veterans Affairs Medical Center, Miami, FL 33125, USA; 2 Division of Gerontology & Geriatric Medicine, Department of Medicine, University of Miami Miller School of Medicine, Miami FL 33136, USA; 3 Sylvester Comprehensive Cancer Center, University of Miami Miller School of Medicine, Miami FL 33136, USA

## Abstract

**Background:**

NF-κB is a transcription factor that promotes inhibition of apoptosis and resistance to chemotherapy. It is commonly believed that inhibition of NF-κB activity can increase sensitivity of cancer cells to chemotherapy. However, there is evidence that NF-κB activation can sensitize cells to apoptosis and that inhibition of NF-κB results in resistance to chemotherapy. In prostate cancer, it is not clear in the different cell types (androgen-dependent and castration-resistant) if activation or inhibition of NF-κB is required for stimulation of apoptosis by chemotherapy.

**Results:**

Our data indicate that the response of prostate cancer (PC) cells to the antimitotic drugs docetaxel (Doc) and 2-methoxyestradiol (2ME2) is dependent on the levels of NF-κB activity. In androgen-dependent LNCaP cells, Doc and 2ME2 treatment increased the low basal NF-κB activity, as determined by Western blot analysis of phospho-IκBα/p65, NF-κB promoter reporter assays, and p65 localization. Treatment of LNCaP cells with parthenolide, a pharmacologic inhibitor of NF-κB, or introduction of dominant-negative IκBα, or an shRNA specific for p65, a component of the NF-κB heterodimer, blocked apoptosis induced by Doc and 2ME2. In castration-resistant DU145 and PC3 cells, Doc and 2ME2 had little effect on the high basal NF-κB activity and addition of parthenolide did not enhance cell death. However, the combination of Doc or 2ME2 with betulinic acid (BA), a triterpenoid that activates NF-κB, stimulated apoptosis in LNCaP and non-apoptotic cell death in DU145 and PC3 cells. Increased sensitivity to cell death mediated by the Doc or 2ME2 + BA combination is likely due to increased NF-κB activity.

**Conclusions:**

Our data suggest that the combination of antimitotic drugs with NF-κB inhibitors will have antagonistic effects in a common type of PC cell typical of LNCaP. However, combination strategies utilizing antimitotic drugs with BA, an activator of NF-κB, will universally enhance cell death in PC cells, notably in the aggressive, castration-resistant variety that does not respond to conventional therapies.

## Introduction

NF-κB, originally discovered as a transcription factor that regulates the immune system, is now known to be widely expressed in almost all cells and mediates multiple signaling pathways including cell proliferation and survival [1]. A common form of NF-κB is a heterodimer consisting of p65 (RelA) and p50 proteins that exists as an inactive IκBα-bound form in the cytoplasm of unstimulated cells. Activation of the NF-κB pathway by a variety of inducers including cytokines, growth factors, UV light and DNA-damaging drugs often occurs by increasing the phosphorylation of IκBα by the IκB kinase (IKK) complex. This results in ubiquitination and rapid degradation of IκBα by the 26S proteasome, allowing for the increase of nuclear NF-κB DNA binding activity and transcriptional activation of its target genes, including Inhibitor of Apoptosis (IAP) family members XIAP, IAP-1, IAP-2 and anti-apoptotic Bcl-2 family members Bcl-2, Bcl-xL [1]. Therefore, NF-κB activation is often associated with increased survival of cancer cells and resistance to chemotherapy. Accordingly, there are several candidate inhibitors of NF-κB activity that are in development as anti-cancer therapy [2-4].

However, the role of NF-κB in cancer progression and in anti-cancer therapeutics is complex, as there is also evidence to suggest that NF-κB activation can sensitize cells to apoptosis [5,6]. For example, inhibition or loss of NF-κB activity blocks p53-mediated apoptosis, suggesting that inhibition of NF-κB in p53 positive tumors may weaken the therapeutic response [7]. Furthermore, activation of NF-κB by UV light and doxorubicin converts it into an active repressor of the anti-apoptotic genes XIAP and Bcl-xL [8]. Additional evidence supporting a pro-apoptotic role for NF-κB in cancer chemotherapy comes from the observation that the retinoid-related compounds 3-Cl-AHPC and CD437 require activation of NF-κB in order to induce apoptosis in DU145 and PC3 castration-resistant prostate cancer (CRPC) cells [9,10]. Exposure of CRPC cells to 3-Cl-AHPC or CD437 enhances the expression of the pro-apoptotic Death Receptor (DR) 4 and 5 genes. An NF-κB binding site located in intron 1 of the DR5 promoter is important for positive regulation by NF-κB [11]. Activation of NF-κB by betulinic acid (BA), a naturally occurring pentacyclic triterpenoid small molecule with anticancer properties, is also required for induction of apoptosis in tumor cells [12].

The clinical progression of prostate cancer (PC) involves the transition from androgen-dependent cancer, which can be successfully treated with androgen-ablation therapy, to a castration-resistant cancer with few treatment options [13]. One of the critical factors in the progression to CRPC is the increased activity of NF-κB and its promotion of apoptotic inhibition [14-16]. It is not clear in the different types of PC cells (androgen-dependent and castration- resistant) whether activation or inhibition of NF-κB is required for stimulation of apoptosis by chemotherapy. Since PC consists of a heterogeneous mixture of cell types, it is important to better understand the mechanisms of the effect of chemotherapy on NF-κB activity in different PC cell lines in order to increase therapeutic response [17,18].

In this report, we investigate the effects of the antimitotic drugs docetaxel (Doc) and 2-methoxyestradiol (2ME2) on NF-κB activity and induction of cell death in androgen-responsive and castration-resistant PC cell lines. Doc is now one of the most effective anti-cancer drugs and is FDA approved for the treatment of prostate, breast, gastric, head and neck, and non-small cell lung cancers [19,20]. 2ME2, an endogenous metabolite of estradiol, is being investigated in clinical trials as an anti-cancer agent [21]. Both Doc and 2ME2 have been reported to increase NF-κB activity in tumor cell lines including PC, but whether this stimulates or antagonizes apoptosis appears to be dependent on the specific tumor cell type [22,23].

Studies have shown constitutive NF-κB DNA binding and transcriptional activity in DU145 and PC3 CRPC cells but not in androgen-dependent LNCaP cells [24-26]. Our data indicated that both Doc and 2ME2 increased NF-κB activity in LNCaP cells and that inhibition of NF-κB was able to block treatment-induced apoptosis. Doc and 2ME2 treatment had little effect on NF-κB activity in DU145 and PC3 cells and the addition of an NF-κB inhibitor did not stimulate cell death in these cells. In contrast, addition of BA increased NF-κB activity and stimulated Doc- and 2ME2-mediated apoptosis in LNCaP and caspase-independent cell death in DU145 and PC3 cells.

## Materials and methods

### Reagents

2ME2 was obtained from EntreMed, Inc. and Doc from Aventis Pharmaceuticals. Parthenolide and 4'-6-Diamidino-2-phenylindole (DAPI) were purchased from Calbiochem; BA from Calbiochem, Biomol, or AG Scientific; Trypan blue (0.4%) from Invitrogen; and Coomassie blue from EMD Chemicals, Inc.

### Cell Culture

Human PC cell lines LNCaP, DU145, and PC3 were obtained from the American Type Culture Collection [27]. LN-AI is a castration-resistant subline of LNCaP, which was spontaneously derived in our laboratory [28]. These cells express androgen receptor (AR) and prostate-specific antigen (PSA), similar to LNCaP. DU145 and PC3 cells do not express AR or PSA. All cells were maintained in RPMI 1640 medium (Invitrogen) with 5% fetal bovine serum (Hyclone), 100 U/ml penicillin, 100 μg/ml streptomycin, and 0.25 μg/ml amphotericin (Invitrogen). Media for LN-AI/dnI clones 7, -20, and LN-AI/neo cells also contained 200 μg/ml G418 (Invitrogen).

### Drug Treatments

PC cells were cultured in media containing 2ME2 (5 μM), Doc (1 nM), parthenolide (10 μM), BA (10 μM) or DMSO control for varying times (24-72 h). In all the experiments, adherent and non-adherent cells were pooled for further analysis.

### Western Blot Analysis

Preparation of total protein lysates was done as previously described [28]. Preparation of nuclear extracts was done using NE-PER nuclear extraction reagents as per manufacturer's instructions (Pierce Biotechnology). After separation of 25-50 μg protein by SDS-PAGE, proteins were transferred by electrophoresis to Immobilon-P membrane and incubated in 5% nonfat dry milk, TBS, and 0.1% Tween-20 for 1 h. Antibodies specific for phospho-(Ser32/36) IκBα (9246), IκBα (9242), phospho (Ser536)-p65 (3031), cleaved PARP (Asp 214), and XIAP (2042) from Cell Signaling; p65 (C-20), p53 (DO-1), and AIF/N-terminus (E-1) from Santa Cruz Biotechnology; and AIF/C-terminus (A7549) from Sigma-Aldrich were diluted 1/1,000-1/3,000 in 5% nonfat dry milk, TBS, and 0.1% Tween-20 and incubated overnight at 4°C. Membranes were washed in TBS and 0.1% Tween-20 and incubated with the appropriate horseradish peroxidase-conjugated secondary antibody (1/3,000 dilution; Santa Cruz) for 1 h, washed in TBS and 0.1% Tween-20, and analyzed by exposure to X-ray film using enhanced chemiluminescence plus (ECL plus, GE Healthcare Bio-Sciences Corp). Staining of total protein with Coomassie blue was used as a protein loading control. X-ray films were scanned using an Epson Perfection 2450 Photo scanner.

### NF-κB Reporter Assay

To measure NF-κB transcription activity, we used a plasmid containing the luciferase reporter gene regulated by four copies of NF-κB cis-acting elements linked to TATA box from the thymidine kinase promoter (NF-κB-TA/luc; Clontech). TA/Luc is the negative control plasmid without NF-κB elements. Plasmids were co-transfected with CMV/β-galactosidase (Clontech) into LNCaP and PC3 cells using FuGene 6 HD transfection reagent (Roche), as previously described [29]. After 24 h, transfected cells were grown in the presence or absence of 5 μM 2ME2 for 24 and 72 h and the luciferase and β-gal activities determined. The NF-κB luciferase activity relative values as light units/β-gal were divided by TA/luc values and expressed as fold above TA/luc from 3 independent experiments done in duplicate.

### Fluorescence Immunocytochemistry

Adherent and non-adherent cells were harvested, fixed in formalin for 5 min, applied to slides by smearing, air dried, rinsed with PBS, and blocked with M.O.M. (Vector Laboratories) or goat serum for 20 min. For double immunofluorescence, after first immunostaining for p65 (1:50 dilution; 30 min), we used biotinylated anti-rabbit IgG (1/200 dilution; Vector Laboratories) for 20 min, fluorescein Avidin DCS (1/300 dilution; Vector Laboratories) for 5 min., followed by avidin/biotin blocking for 15 min, immunostaining with nucleolin (H-250; Santa Cruz Biotechnology) for 30 min, biotinylated anti-rabbit IgG, Texas Red DCS, and mounting media with DAPI stain (Vector Laboratories). AIF was immunostained using AIF/N-terminus (1:25 dilution), biotinylated anti-mouse IgG (1/200 dilution; Vector Laboratories), Fluorescein Avidin DCS, and mounting media with propidium iodide (PI) (Vector Laboratories). Color images were acquired using a Nikon Eclipse 90i fluorescence microscope with FITC/Texas Red filters and merged using Adobe Photoshop 7.

### DAPI Apoptosis and Trypan Blue Exclusion Assays

The DAPI staining apoptosis assay was done as previously described [28]. Changes in apoptosis in cells treated with drugs were determined as percentage of apoptotic cells (densely stained and fragmented chromatin) from 3-6 independent experiments done in duplicate. Minimal apoptosis was detected in control treated cells (<0.5%). For the trypan exclusion assay, treated and control prostate cancer cells were harvested, resuspended in growth media, diluted 1:1 in 0.4% trypan blue, dead blue and live non-blue cells immediately counted using a hemacytometer, and the % dead blue cells determined from at least 3 independent experiments done in duplicate.

### Annexin-FITC/Propidium Iodide (PI) Flow Cytometry

For the annexin apoptosis assay, we used the ApoAlert Annexin V-FITC Apoptosis kit (Clontech). LN-AI and DU145 cells were resuspended in binding buffer followed by the addition of annexin V-FITC and PI. After 20 min., cells were analyzed by flow cytometry using a Coulter XL flow cytometer and the percentage of annexin+ and PI+ cells determined using WinMDI version 2.8.

### Stable Transfection of Dominant Negative IκBα

To inhibit endogenous NF-κB activity, we obtained the pCMV-IκBαM plasmid (Clontech) expressing dominant negative IκBα containing Ser to Ala mutations at positions 32 and 36, which cannot be phosphorylated and degraded [30]. LN-AI cells (90% confluent) were co-transfected with pCMV-IκBαM and pCMVneo (for drug selection) using FuGene 6 HD following the manufacturer's instructions. The negative control was transfection with pCMVneo alone. Cells were initially grown in media with 400 μg/ml G418 (Invitrogen), colonies selected, and clones that express dominant negative-IκBα (dnI) compared to pCMVneo negative control cells clones were identified by Western blot (migrates faster than endogenous wild type IκBα).

### Lentiviral Transduction of LNCaP and DU145 with shRNA Against p65 and AIF

The shRNA design, lentivirus production and infection were done as previously described [31]. The following DNA oligonucleotides (Operon Technologies) targeting p65 and AIF were cloned into pLKO.1 lentivirus vector: shp65-1: GGCGGATTGAGGAGAAACGTAAACTCG AGTTTACGTTTC TCCTCAATCCGTTTTTG; shp65-2: CCGGCCTGAGGCTATAACTCG CCTACTCGAGTAGGCGA GTTATAGCCTCAGGTTTTTG; shAIF-1: CCGGCCTGGAAA TAGACTCAGATTT CTCGAGAAATCTGAGTCTATTTCCAGGTTTTTG; shAIF-2: CCGG CTGCATGCTTCTACGATATAACTCGAG TTATATCGTAGA AGCATGCAGTTTTTG. The control shRNA was targeted against GFP. LNCaP/shp65-2, LNCaP/shAIF-2, LNCaP/shGFP, DU145/sh p65-1, DU145/shAIF-1, and DU145/shGFP were further analyzed.

### Real Time Quantitative Polymerase Chain Reaction (RT-qPCR)

RNA was isolated from prostate cancer cells using QIAshredder and RNeasy miniprep kit (Qiagen Inc.). All RNAs were treated with RNase-free DNase (Ambion) to remove possible DNA contamination. The following DNA oligonucleotides (Operon Technologies) were used for RT-qPCR: AIFsh sense 5'-TCATGCCCACTGTCCTGTAAGT-3' and antisense 5'-CCATGG TCCAGTTGCTGAGGT-3' (239 amplicon) [32]; IκBα sense 5'-CTCCGAGACTTTCGAGG AAATAC-3' and antisense 5'-GCCATTGTAGTTGGTAGCCTTCA-3' (135 amplicon) [33]; A20 sense 5'-AAGCTGTGAAGATACGGGAGA-3' and antisense 5'-CGATGAGGGCTTT GTGGATGAT-3' (159 amplicon) [33]; DR5 sense 5'-AAGACCCTTGTGCTCGTTGT-3' and antisense 5'-AGGTGGACACAATCCCTCTG-3' (144 amplicon) [33]; and the reference gene ribosomal protein, large, P0 (RPL0) sense 5'-GCAATGTTGCCAGTGTCTG-3' and antisense 5'-GCCTTGACCTTTTCAGCAA-3' (141 amplicon) [34]. cDNA was synthesized with the iScript cDNA synthesis kit (Bio-Rad) and qPCR with Brilliant II Sybr Green QPCR Kit at 30 sec at 95°C, 1 min at 55°C, and 30 sec at 72°C for 40 cycles using a MX3005 qPCR system (Stratagene). Crossing point values from logarithmic amplification profiles for target genes were divided by values from the RPL0 reference gene from RNA samples and presented as fold above control treated cells analyzed five times from three independent experiments. Each product was confirmed for the expected size by agarose gel electrophoresis.

### Statistical Analysis

Statistical differences between drug-treated and control PC cells were determined by two-tailed Student's *t*-test with *P *< 0.05 considered significant.

## Results

To evaluate the effect of the antimitoic drugs 2ME2 and Doc on NF-κB activity in human PC cells, we used androgen-dependent LNCaP and castration-resistant LN-AI, DU145, and PC3 cells. LNCaP and LN-AI cells contain wild-type p53 and exhibit higher sensitivity to 2ME2 and Doc apoptosis relative to DU145 and PC3, which are p53 mutated or null and thus are more resistant to apoptosis [27,28,35-37].

### Antimitotic Drugs Activate NF-κB in LNCaP Cells

**Figure 1:**
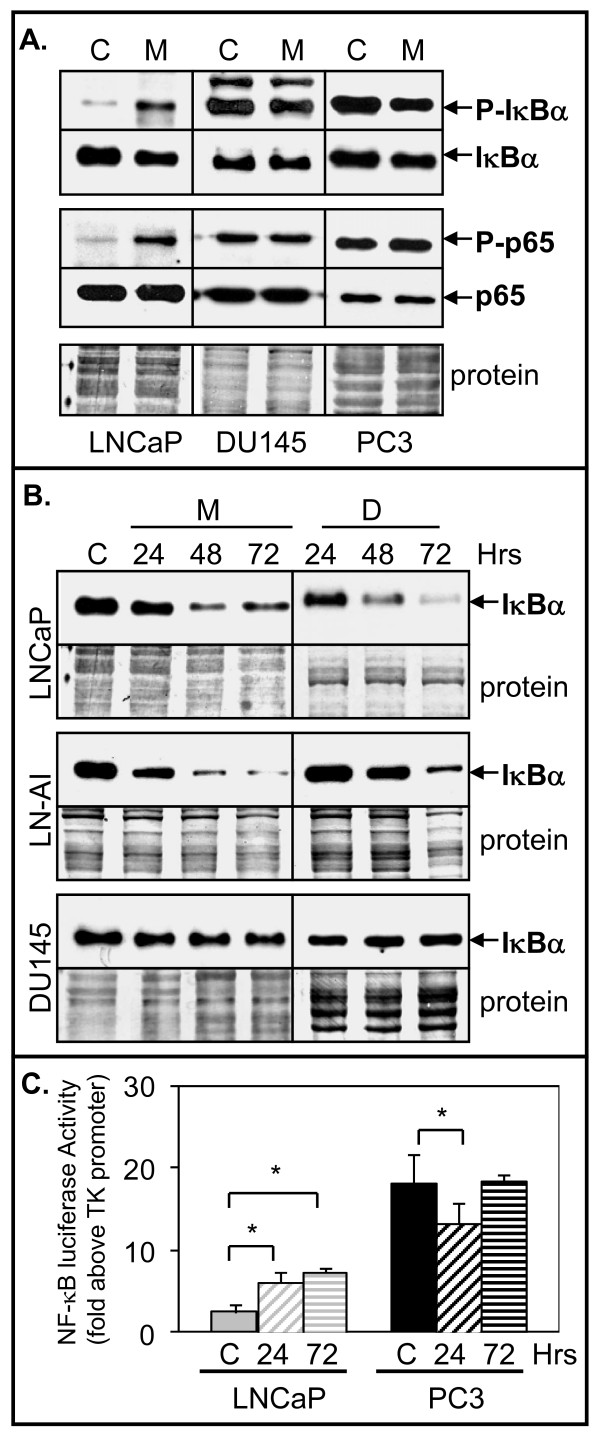
**2ME2 increases NF-κB activity in LNCaP but not in DU145 or PC3 cells**. **A**. Western blot analysis showing that treatment of LNCaP but not in DU145 or PC3 cells with 5 μM 2ME2 (M) for 24 h increases phospho (P)-IκBα and P-p65 compared to control (C). Total levels of IκBα and p65 do not change, except for a decrease of IκBα in LNCaP. Coomassie blue stain of total protein is loading control. **B**. Western blot analysis showing that 2ME2 (M; 5 μM) and Doc (D; 1 nM) decrease IκBα levels at 48 and 72 h in LNCaP and LN-AI compared to control (C) cells. In DU145 cells, M and D did not decrease IκBα levels. **C**. Luciferase reporter assay showing that 2ME2 increases NF-κB activity (fold above TK promoter) in LNCaP cells at 24 h and remains high at 72 h compared to control (C) cells. In PC3 cells, the basal NF-κB activity is 7.3-fold higher compared to LNCaP cells and 2ME2 reduces activity after 24 h. After 72 h, NF-κB activity is similar to control cells. n = 6, three independent experiments; *, *P *< 0.02.

To determine the effect of 2ME2 and Doc on NF-κB, we analyzed phosphorylation of IκBα and of p65 at the serine 536 position, which can enhance the transcriptional activity of NF-κB and lower its affinity to IκBα [1]. Western blot analysis of LNCaP cells treated with 5 μM 2ME2 for 24 h resulted in increased phospho-IκBα (decrease in total IκBα) and phospho-p65 (no change in total p65) relative to control treated cells (Fig. 1A). In DU145 and PC3 cells, which unlike LNCaP cells have constitutively active NF-κB, there were no differences in phospho-IκBα or phospho-p65 in 2ME2-treated cells relative to control cells (Fig. 1A). In addition, total IκBα protein decreased in 2ME2 or Doc-treated LNCaP and LN-AI cells but not in DU145 cells (Fig. 1B).

To determine the effect of 2ME2 on NF-κB transcriptional activity, we used a plasmid containing the luciferase reporter regulated by NF-κB cis-acting elements. The results showed that 2ME2 increased NF-κB activity ~2-fold in LNCaP cells at 24 and 72 h compared to control treated cells (Fig. 1C). In contrast, 2ME2 slightly decreased NF-κB activity 1.5-fold in PC3 cells at 24 h but not at 72 h. The results also showed a 7.3-fold higher basal NF-κB activity in PC3 compared to LNCaP cells.

### Nucleolar Localization of p65 in LNCaP cells Treated with Anitmitotic Drugs

**Figure 2:**
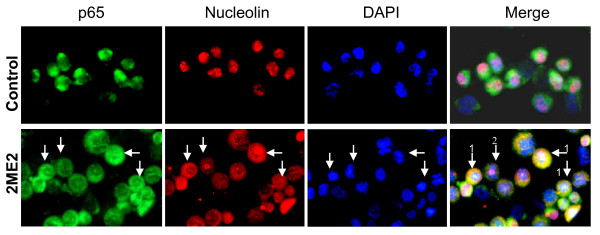
**2ME2 increases p65 nucleolar localization in LNCaP cells**. Double immunofluorescence showing localization of p65 to the nucleolus in LNCaP cells treated with 2ME2 for 24 h. In control LNCaP cells, p65 (green) is cytoplasmic, nucleolin (red) is in the nucleolus, DAPI (blue) stains the nucleus, and the merge of p65/nucleolin/DAPI shows no co-localization of p65 and nucleolin or DAPI. In 2ME2 treated LNCaP cells, p65 remains mostly cytoplasmic but some is localized within the nucleus; nucleolin is localized to the cytoplasm and some remains in the nucleolus or nucleus; DAPI shows mitotic cells. The merge of p65/nucleolin/DAPI shows some cells (arrows 1) with co-localized p65/nucleolin within the nucleolus (white) and within the cytoplasm (yellow). Also shown is a cell (arrow 2) with no co-localization of p65 and nucleolin. (×200).

We used fluorescence immunocytochemistry to determine if p65 localizes to the nucleus after 2ME2 treatment of LNCaP cells. In control cells, p65 localized in the cytoplasm but after 24 h treatment with 5 μM 2ME2, some cells appeared to have p65 localization to the nucleolus, similar to a previous report [38]. Double fluorescence immunocytochemistry confirmed the presence of several cells with localization of p65 to the nucleolus, as revealed by merged images of the nucleolar marker nucleolin (Texas Red) with p65 (FITC), and DAPI nuclear stain (blue) (Fig. 2). There was also co-localization of p65 and nucleolin in the cytoplasm (yellow), likely due to the disruption of the nucleolus and nuclear membrane by antimitotic drugs. The nucleolus is normally disassembled during mitosis and reassembled after cell division [39]. These results suggest that in LNCaP cells treated with antimitotic drugs, p65 can localize to the nucleolus, which has previously been shown to be important in increasing apoptosis in colon cancer cells treated with aspirin [38].

### NF-κB Inhibition Blocks Apoptosis Induced by Antimitotic Drugs

**Figure 3:**
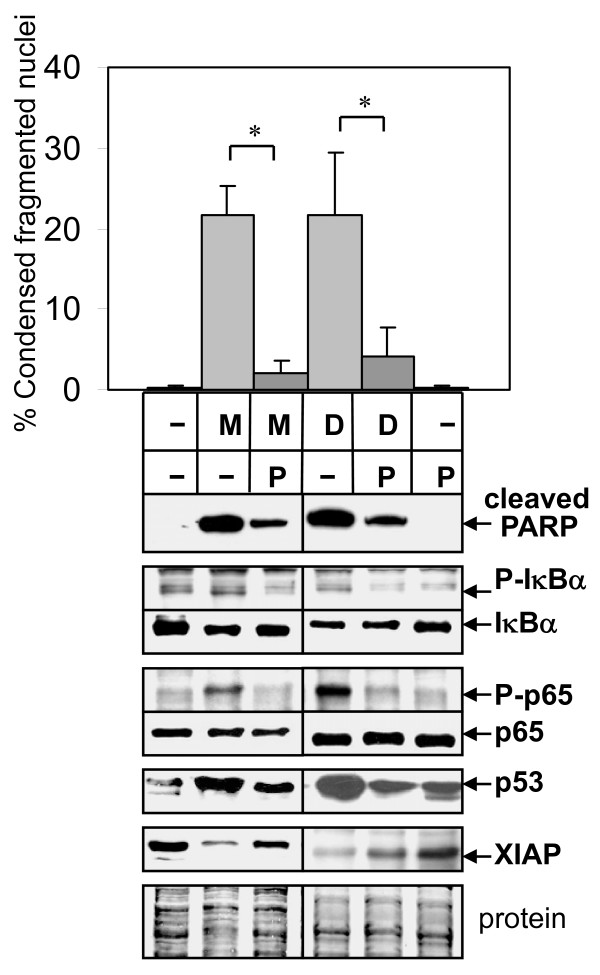
**NF-κB inhibitor parthenolide blocks 2ME2 and Doc-mediated apoptosis in LNCaP cells**. DAPI apoptosis analysis (% condensed fragmented nuclei) showing that parthenolide (P; 10 μM) blocks 2ME2 (M; 5 μM) and Doc (D; 1 nM) apoptosis in LNCaP (72 h) cells. n = 7, four independent experiments; *, *P *< 6 × 10^-4^. Western blot analysis shows that P inhibits the M and D increase in cleaved PARP (72 h), P-IκBα/IκBα (24 h), P-p65/p65 (24 h), p53 (72 h), and the M/D decrease in XIAP (72 h). Coomassie blue stain of total protein is loading control.

To determine whether 2ME2- or Doc-mediated activation of NF-κB in LNCaP cells is important for stimulating apoptosis, we used the NF-κB inhibitor parthenolide [40]. Treatment of LNCaP cells with 10 μM parthenolide lowered apoptosis induced by 2ME2 or Doc, as determined by the DAPI apoptosis assay and decreased levels cleaved PARP protein (Fig. 3). Parthenolide lowered the 2ME2- or Doc-mediated increase in phospho-IκBα and phospho-p65, suggesting inhibition of NF-κB activity. These results indicate that 2ME2- or Doc-mediated increase in NF-κB activity is important for induction of apoptosis in LNCaP cells. Similar results were obtained in LN-AI cells (result not shown).

To further investigate molecular changes involved in why inhibition of NF-κB reduced 2ME2- or Doc-mediated apoptosis, we analyzed the expression of p53 and XIAP. p53, the most commonly mutated gene in human cancers, can mediate the apoptosis response to chemotherapy [41]. Overexpression of IAP family members such as XIAP blocks apoptosis and increases drug resistance [42]. Similar to our previous results, 2ME2 and Doc increased p53 and decreased XIAP proteins in LNCaP and LN-AI cells [28,35]. However, parthenolide blocked the 2ME2 and Doc-induced changes in p53 and XIAP relative to control levels (Fig. 3). These results suggest that the 2ME2- or Doc-mediated increase in NF-κB activity correlates with increased p53 and decreased XIAP, conditions that favor the induction of apoptosis.

### Dominant Negative IκBα and p65 Knockdown Inhibit 2ME2 and Doc Apoptosis

**Figure 4:**
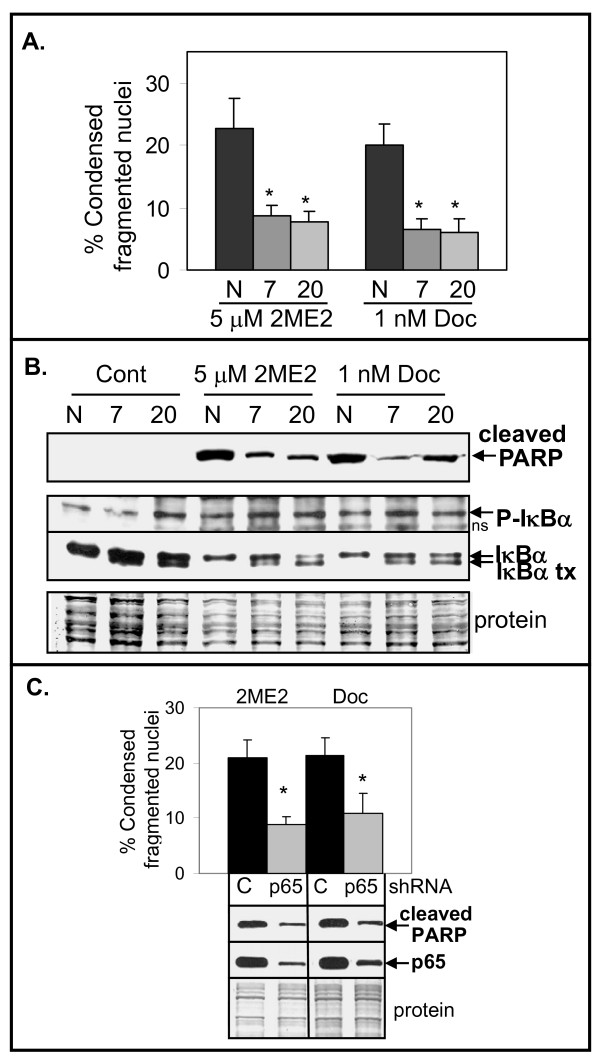
**Inhibition of NF-κB with dominant negative IκBα (dnI) or p65 shRNA decreases apoptosis induced by 2ME2 and Doc**. **A**. DAPI analysis showing that LN-AI/dnI clones 7 and 20 undergo less apoptosis (% condensed fragmented nuclei) when treated with 2ME2 (M; 5 μM) or Doc (D; 1 nM) for 48 h compared to LN-AI/neo negative control (N). n = 8, four independent experiments; *, *P *< 5 × 10^-5^. **B**. Western blot analysis showing less cleaved PARP in LN-AI/dnI clones 7 and 20 compared to LN-AI/neo (N) cells treated with M or D for 48 h. No cleaved PARP was detected in control (Cont) treated cells. Endogenous phospho (P)-IκBα increases in LN-AI/dnI-7 and neo (N) cells but not in LN-AI/dnI-20 cells when treated with M or D for 24 h. Total levels of endogenous IκBα decrease in all cells treated with M or D compared to Cont treated cells. Transfected (tx) dnI protein (migrates slightly faster than endogenous IκBα) remains in LN-AI/dnI clones 7 and 20 but is not detected in LN-AI/neo cells. ns, non-specific band. Coomassie blue stain of total protein is loading control. **C**. DAPI and Western blot analysis showing less apoptosis and cleaved PARP in p65 knockdown LNCaP/shp65-2 (p65) compared to control LNCaP/GFP (C) cells treated with 2ME2 or Doc for 72 h. n = 6, three independent experiments; *, *P *< 5 × 10^-3^.

To further determine if there is a role for activation of NF-κB in 2ME2- or Doc-mediated apoptosis, we isolated stably transfected LN-AI clones expressing dominant negative IκBα (dnI) [30]. LN-AI/dnI clones 7 and 20 and negative control LN-AI/neo cells were treated with 2ME2 or Doc for 48 h and the effect on apoptosis determined by DAPI and cleaved PARP protein levels. Results showed decreased apoptosis in LN-AI/dnI-7 and 20 compared to LN-AI/neo cells (Fig. 4). As expected, there was an increase in endogenous phospho-IκBα in all cells treated with 2ME2 or Doc for 24 h but phospho-dnI was not detected (faster migrating band in total IκBα blot; Fig. 4B). Similar results were obtained in LNCaP cells stably expressing dnI (result not shown) or shRNA targeting p65 (Fig. 4C). These results further support an important role for NF-κB activation-mediated increase in apoptosis by treatment with 2ME2 or Doc in LN-AI and LNCaP cells.

### Betulinic Acid (BA), an NF-κB Activator, Stimulates Cell Death in All Prostate Cancer Cells Treated with Antimitotic Drugs

**Figure 5:**
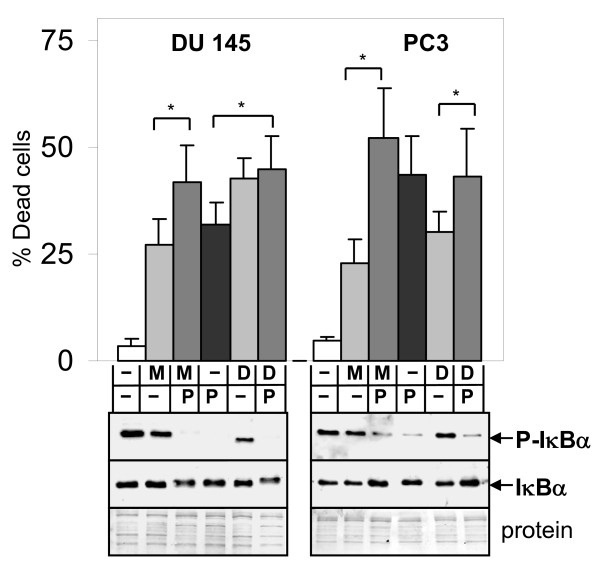
**NF-κB inhibitor parthenolide has little effect on 2ME2 or Doc cell death in DU145 and PC3 cells**. Trypan blue exclusion assay showing that 72 h treatment with parthenolide (P; 10-20 μM) does not significantly increase 2ME2 (M; 5 μM) or Doc (D; 1 nM) cell death (% dead cells), with the exception of M vs. MP and P vs. DP in DU145 (*, *P *< 0.02) and M vs. MP and D vs. DP in PC3 cells (*, *P *< 0.01). Cell death in control treated cells is < 5%. n = 4-5, three independent experiments. Western blot analysis at 24 h shows that P inhibits P-IκBα/IκBα in DU145 and PC3 cells. Coomassie blue stain of total protein is loading control.

**Figure 6:**
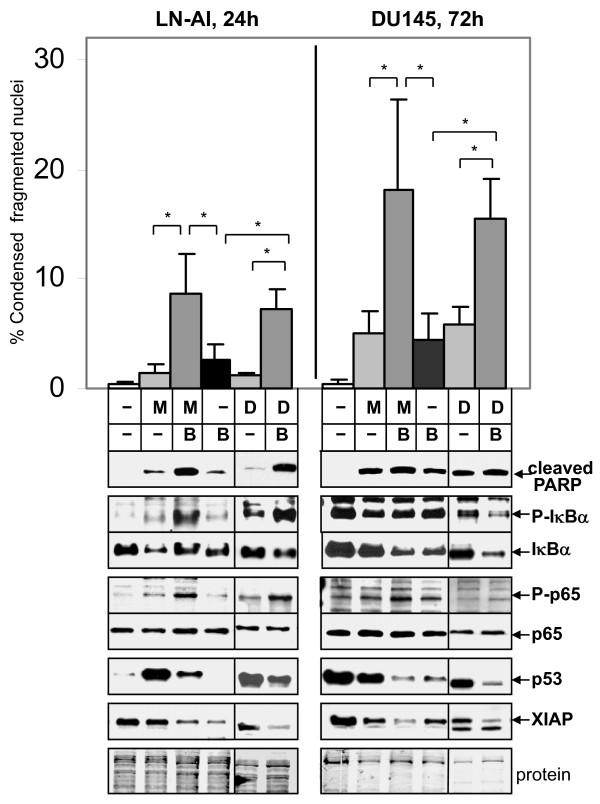
**NF-κB activator BA stimulates 2ME2 and Doc-mediated apoptosis in LN-AI and DU145 cells**. DAPI apoptosis assay (% condensed fragmented nuclei) showing that BA (B; 10 μM) increases 2ME2 (M; 5 μM) and Doc (D; 1 nM) apoptosis (MB and DB) in LN-AI (24 h) and DU145 (72 h) compared to M, B, and control cells. n = 6-8, three to four independent experiments; *, *P *< 0.004. Western blot analysis shows greater cleaved PARP in MB and DB compared to M, D, or B alone in LN-AI but not in DU145 cells. MB and DB increases P-IκBα/IκBα and P-p65/p65 in LN-AI and DU145 cells at 24 h compared to M, D, B, and control cells, suggesting activation of NF-κB. MB and DB decreases IκBα, p53 and XIAP compared to M and D alone; B alone also decrease IκBα, p53, and XIAP compared to control cells. Coomassie blue stain of total protein is loading control.

**Figure 7:**
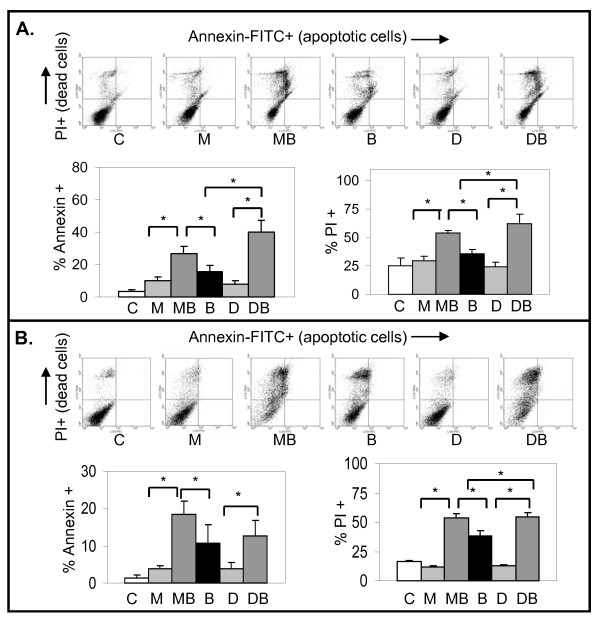
**2ME2/Doc + BA combination increases annexin+ apoptosis and PI+ cell death greater than either alone**. Flow cytometric analysis of annexin-FITC (apoptotic) and PI (dead) stained LN-AI (A) and DU145 (B) cells after treatment with M, D, B, MB, DB, and control (C) for 24 h (LN-AI) and 48 h (DU145). The annexin+ and PI+ cells were measured from three independent experiments done in duplicate (n = 6). *, *P *< 0.04.

Our results showed that the NF-κB inhibitor parthenolide antagonized apoptosis mediated by antimitotic drugs in LNCaP and LN-AI cells. In DU145 or PC3 cells, parthenolide had little effect on 2ME2- or Doc-mediated cell death, as shown by the trypan blue exclusion assay (Fig. 5). Thus, we determined whether addition of an NF-κB activator could increase apoptosis by antimitotic drugs in all types of PC cells. BA is a natural pentacyclic triterpenoid proposed to activate NF-κB [12]. Combination of 2ME2 or Doc with 10 μM BA increased apoptosis in LN-AI cells compared to the single drugs, as determined by DAPI, cleaved PARP protein levels, and annexin V-FITC/PI flow cytometry (Figs. 6, 7). BA further increased phospho-IκBα and phospho-p65 and decreased total IκBα compared to 2ME2 or Doc alone in LN-AI and DU145 cells, suggesting enhancement of NF-κB activity (Fig. 6). Similar results were obtained in LNCaP cells (results not shown). In addition, BA increased NF-κB DNA binding in LNCaP and DU145 cells as determined by EMSA (result not shown). In DU145 and PC3 cells, despite increased fragmented and condensed nuclei (DAPI), BA +2ME2 or Doc did not increase cleaved PARP (substrate for active caspase) (Fig. 6 and not shown), suggesting caspase-independent cell death.

**Figure 8:**
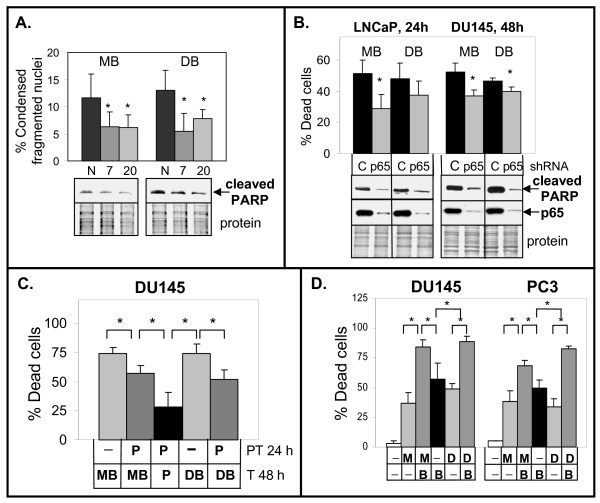
**Activation of NF-κB by BA is important for increasing apoptosis/cell death in combination with 2ME2 or Doc**. **A**. DAPI analysis showing that LN-AI/dnI clones 7 and 20 undergo less apoptosis (% condensed fragmented nuclei) when treated with MB or DB for 24 h compared to LN-AI/neo negative control (N). n = 6, three independent experiments; *, *P *< 0.04. Western blot analysis showing less cleaved PARP in LN-AI/dnI clones 7 and 20 compared to LN-AI/neo (N) cells treated with MB or DB for 24 h. **B**. Trypan blue exclusion and Western blot analysis showing that knockdown of p65 in LNCaP/shp65-2 and DU145/shp65-1 lowers cell death (n = 6, three independent experiments; *, P < 0.003) and cleaved PARP when treated with MB or DB and compared to control (C) treated LNCaP/shGFP and DU145/shGFP cells. **C**. Trypan blue exclusion assay showing that DU145 cells pretreated (PT) with NF-κB inhibitor parthenolide (Pa; 10 μM) for 24 h then treated (T) with MB or DB + Pa for 48 h underwent less cell death (% dead cells) compared to cells not pretreated with Pa and treated with MB or DB. Also shown are cells pretreated and treated with Pa alone. n = 6-7, four independent experiments. *, P < 0.002. **D**. Trypan blue exclusion assay showing increase in cell death in DU145 and PC3 cells treated for 72 h with 2ME2 + BA (MB) or Doc + BA (DB) compared to BA (B; 10 μM), 2ME2 (M; 5 μM), Doc (D; 1 nM) alone and control cells. n = 5-6, three independent experiments; *, *P *< 0.004.

Further supporting a role for activation of NF-κB in the 2ME2/Doc + BA combination is the observation that the LN-AI/dnI clones underwent less apoptosis compared to the LN-AI/neo negative control cells (Fig. 8A). Knockdown of p65 in LNCaP and DU145 also lowered cell death and cleaved PARP (Fig. 8B). In addition, pretreatment of DU145 cells with parthenolide (10 μM) for 24 h followed by 2ME2/Doc + BA/parthenolide lowered cell death compared to DU145 cells treated without parthenolide (Fig. 8C). Furthermore, PC cells treated with BA alone or in combination with 2ME2 or Doc decreased IκBα and XIAP protein levels (Fig. 6). These results suggest that enhancement of NF-κB activity by BA plays a role in increasing cell death in PC cells treated with antimitotic drugs.

Because cleaved PARP levels are not increased in DU145 or PC3 cells treated with the 2ME2/Doc + BA combination, we assessed whether there were any differences in total cell death by the trypan blue exclusion assay. Results indicated a greater extent of cell death in the 2ME2/Doc + BA combination compared to 2ME2, Doc, or BA treatment alone (Fig. 8D). This result further suggests that BA increases caspase-independent cell death in DU145 and PC3 cells.

### Apoptosis-Inducing Factor (AIF) Increases Cell Death by 2ME2/Doc + BA

**Figure 9:**
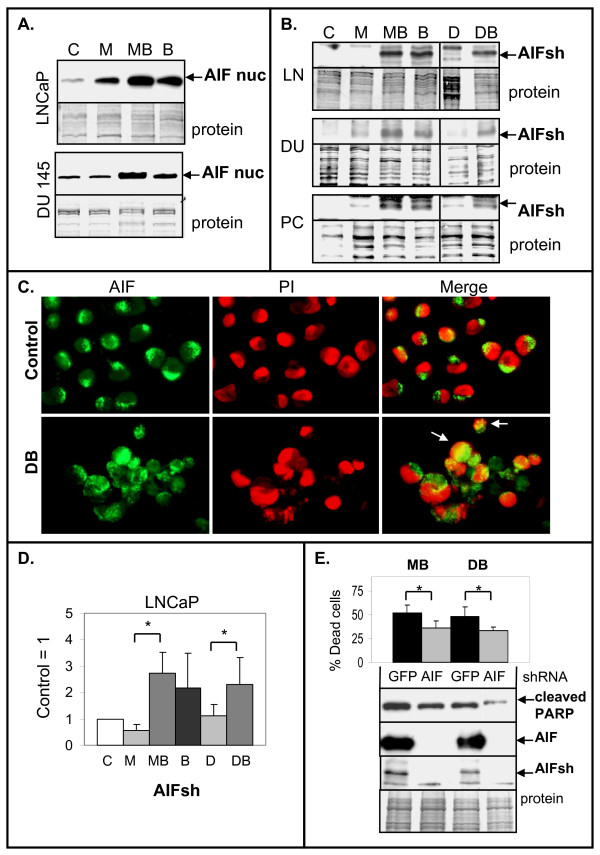
**BA increases AIF nuclear localization and expression of AIFsh in PC cells**. **A**. Western blot analysis showing increase in nuclear AIF (57 kd) in LNCaP (48 h) and DU145 (72 h) cells treated with MB compared to M, B, and control (C) cells. **B**. Western blot analysis of total protein lysates showing increase in AIFsh (35 kd) in MB, DB, and B treated LNCaP (48 h), DU145, and PC3 (72 h) compared to M, D, or C. Coomassie blue stain of total protein is loading control. **C**. Immunofluorescence showing increase in nuclear localization of AIF in DU145 cells treated with DB compared to C cells (72 h). In control DU145 cells, AIF (green) is cytoplasmic and the merge with nuclear PI stain (red) shows little nuclear localization. In DB treated DU145 cells, the merge of AIF and PI shows some nuclear localization (arrows; yellow). (×200). **D**. RT-qPCR analysis showing increase in AIFsh mRNA in LNCaP cells treated 48 h with MB, DB, or B alone compared to M or D alone. AIFsh mRNA levels were relative to C = 1. n = 5, three independent experiments; *, *P *< 0.05. **E**. Knockdown of AIF reduces cell death in LNCaP cells treated with MB and DB. Trypan blue exclusion assay showing that LNCaP/shAIF-2 (AIF) cells undergo less cell death compared to control LNCaP/shGFP (C) after treatment for 24 h with MB or DB. n = 6, three independent experiments; *, *P *< 0.02. Western blot analysis showing knockdown of AIF and AIFsh proteins in LNCaP/shAIF-2 results in lower cleaved PARP after MB or DB treatment compared to control LNCaP/shGFP cells. Coomassie blue stain of total protein is loading control.

To investigate potential downstream effectors of increased cell death mediated by BA, we analyzed the expression of apoptosis-inducing factor (AIF), the first identified protein involved in caspase-independent cell death [43]. Upon cytotoxic insult, AIF is cleaved, released from the mitochondria to the cytoplasm and translocates to the nucleus where it causes caspase-independent DNA fragmentation and cell death [32,43]. Western blotting indicated that DU145 and LNCaP cells treated with 2ME2/Doc + BA increased nuclear AIF compared to single drug treatment and control (Fig. 9A). Increased nuclear AIF was also detected by immunofluorescent cell staining of DU145 cells treated with 2ME2/Doc + BA and compared to control cells (Fig. 9C).

**Figure 10:**
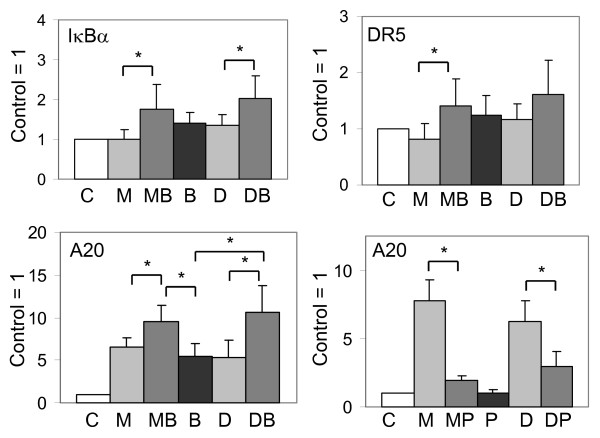
**BA increases and parthenolide decreases expression of NF-κB target genes**. RT-qPCR analysis showing that BA (B; 10 μM) increases expression of IκBα (top left), DR5 (top right), and A20 (bottom left) mRNAs in combination with 2ME2 (M; 5 μM) or Doc (D; 1 nM) (MB, DB) in LNCaP cells (48 h). Parthenolide (P; 10 μM) decreases the M and D increase in A20 (MP, DP) in LNCaP cells (72 h) (lower right panel). mRNA levels are normalized to control (C) = 1. n = 5, three independent experiments. *, *P *< 0.05.

In addition, we investigated the expression of AIFshort (AIFsh) protein, an alternative transcription start site coding for AIF corresponding only to the C-terminal pro-death portion that translocates to the nucleus to induce cell death [32]. Western blot and RT-qPCR analysis showed that LNCaP cells treated with 2ME2/Doc + BA or BA alone increased AIFsh protein and mRNA (2-5-fold) compared to 2ME2 and Doc alone (Fig. 9B, D). IκBα, A20, and DR5 mRNAs, genes known to be regulated by NF-κB [1] also increased in LNCaP cells treated with the 2ME2/Doc + BA combination (Fig. 10). In DU145 and PC3 cells, there was also an increase in AIFsh protein after treatment with 2ME2/Doc + BA or BA alone (Fig. 9B).

Finally, we analyzed if shRNA knockdown of AIF has any effect on cell death induced by the 2ME2/Doc + BA combination in LNCaP and DU145 cells. In LNCaP cells, AIF shRNA reduced both total AIF and AIFsh protein and lowered cell death compared to control LNCaP/shGFP cells (Fig. 9E). In DU145 cells, AIF knockdown also lowered cell death and cleaved PARP by the 2ME2/Doc + BA combination (result not shown). We suggest that the NF-κB activator BA increases expression of AIFsh and stimulates caspase-independent cell death in apoptosis resistant PC cells such as DU145.

## Discussion

Treatment of cancer cells with chemotherapeutic drugs often results in substantial heterogeneity in the response to NF-κB activity. In some cases, NF-κB activation by chemotherapeutic drugs elicits a pro-survival cellular response and combination with inhibitors of NF-κB improves efficacy [1,3,4]. However, depending on the type of drug or cancer cell, activation of NF-κB can elicit a pro-death response [6]. Our results indicated that improving the cell death response to 2ME2 and Doc in PC cells depends on stimulating rather than inhibiting NF-κB activity. In contrast to what was observed with NF-κB inhibitors, combination of 2ME2 or Doc with BA, an activator of NF-κB, increased cell death in androgen-responsive as well as castration-resistant PC cell lines. Therefore, our data suggests that a chemotherapy combination strategy utilizing antimitotic drugs with BA is likely to be a more universally effective chemotherapeutic strategy for PC.

Our data suggest that the combination of antimitotic drugs with NF-κB inhibitors will have antagonistic effects in a common type of PC cell typical of LNCaP and LN-AI. Support for this observation comes from a report demonstrating that bortezomib, a proteasome inhibitor that lowers NF-κB activity by blocking degradation of IκBα, inhibits Doc-induced apoptosis in LNCaP cells [44]. More importantly, recent clinical trials indicate that patients with CRPC have no added benefit from bortezomib above Doc in one study and some antitumor activity in another study [45,46]. It is not yet known, however, if more specific inhibitors of NF-κB in combination with antimitotic drugs will have a better therapeutic effect clinically, especially since constitutive NF-κB activity is very prominent in CRPC [14-16].

Our results are similar to a previous report showing that inhibition of NF-κB with dnI or the NF-κB inhibitor BAY 117082 blocks 2ME2-induced apoptosis in LNCaP cells [23]. Others have shown that 2ME2 can inhibit NF-κB in PC3 and medulloblastoma/glioma cell lines and blocking the Doc increase in NF-κB can enhance apoptosis in a variety of cancer cell lines [47-51]. Overall, the heterogeneity implicated in the NF-κB response to anti-cancer drugs is dependent on the specific type of drug and cancer cell.

In LNCaP and LN-AI cells, the requirement of 2ME2 and Doc to activate NF-κB and increase apoptosis may depend upon the p53 tumor suppressor protein [37]. There is evidence suggesting a link between activation of NF-κB and the ability of p53 to induce apoptosis [7,52]. Our results in LNCaP cells indicated that 2ME2 increased nucleolar localization of p65 (Fig. 2). Nucleolar localization of p65 has been previously reported in colon cancer cells treated with aspirin and a model is proposed that the nucleolus sequesters p65 and inhibits its antiapoptotic functions [38]. Interestingly, the ARF tumor suppressor protein is localized to the nucleolus and after activation by oncogenes can prevent Hdm2 from targeting p53 for degradation and therefore increases the stability of p53 [53]. In addition, ARF can modulate p65 transcriptional activity to repress antiapoptotic genes in a p53 independent manner [54]. One of the effects of antimitotic drugs is the disruption of the nucleolus, the release of ARF, and sequestration of Hdm2, which then leads to the stabilization of p53 and subsequent induction of apoptosis [55]. Our results suggest that investigating the mechanistic basis of p65 nucleolar localization is likely to yield significant insights regarding how to optimize the cytotoxic antitumor action of antimitotic drugs.

It is known that the ability of BA to kill cancer cells occurs by multiple signaling pathways including through activation of NF-κB [12,56]. One potential mechanism for NF-κB activation and increase in apoptosis by BA is the degradation of IκBα and XIAP (Fig. 6). Activation of selective proteasome-dependent degradation of Sp1, 3, and 4 transcription factors controlling the proangiogenic gene VEGF and the antiapoptosis gene survivin by BA has been recently reported [57].

The pro-death effects of BA are independent of p53, which is a desirable characteristic for any agent utilized for the treatment of advanced PC, which frequently lacks functional p53 [58]. Our data show that despite a decrease in p53 protein, BA can still increase apoptosis or cell death in all PC cells (Fig. 6). Our results also suggest that BA combined with 2ME2 or Doc increases cell death in a caspase-independent manner (Figs. 6, 7B, 8D). We suggest that one of the factors that allow DU145 cells to overcome the defect in the apoptosis pathway (mutant p53/null Bax) is the increased nuclear translocation AIF/AIFsh to mediate non-apoptotic cell death.

## Conclusions

Combination chemotherapy is required to further improve the survival of patients with CRPC. A prevailing strategy has been to inhibit the NF-κB response in order to block its pro-survival effect and improve drug efficacy. In this study, we demonstrated that in PC cells such as LNCaP and LN-AI, activation of NF-κB by the antimitotic agents 2ME2 or Doc is important for increasing apoptosis. In addition, when 2ME2 or Doc is combined with an NF-κB activator such as BA, there is effective induction of cell death in all the PC cells analyzed. We are currently investigating whether other NF-κB activators will also mediate increased cell death by antimitotic drugs. The combination of antimitotic agents with NF-κB activators may promote the pro-death responses in a greater variety of PC cells, a requirement for increased therapeutic efficacy.

## Abbreviations

PC: prostate cancer; CRPC: castration-resistant prostate cancer; Doc: docetaxel; 2ME2: 2-methoxyestradiol; DAPI: 4'-6-Diamidino-2-phenylindole; PI: propidium iodide; P: parthenolide; BA: betulinic acid; dnI: dominant negative IκBα; AIF: apoptosis-inducing factor; GFP: green fluorescent protein; DR: death receptor; RT-qPCR: real time quantitative polymerase chain reaction; EMSA: electrophoretic mobility shift assay.

## Competing interests

The authors declare that they have no competing interests.

## Authors' contributions

RP and AP carried out all of the Western blots. TR carried out the immunofluorescence experiments. CPS carried out the cell culture, drug treatments, the transfection/luciferse assays, the DAPI/trypan blue/annexin-PI assays, isolation of LN-AI/dnI clones, and lentiviral transduction of shRNA. AP carried out and analyzed all of the RT-qPCR data. RP, AP, TR, PR, and CPS participated in the design of the experiments and critical review of the manuscript. CPS conceived of the study, coordinated and supervised the project, and wrote and edited the manuscript. All the authors read and approved the final manuscript.
